# Seawater is a reservoir of multi-resistant *Escherichia coli*, including strains hosting plasmid-mediated quinolones resistance and extended-spectrum beta-lactamases genes

**DOI:** 10.3389/fmicb.2014.00426

**Published:** 2014-08-20

**Authors:** Marta S. Alves, Anabela Pereira, Susana M. Araújo, Bruno B. Castro, António C. M. Correia, Isabel Henriques

**Affiliations:** Department of Biology and CESAM, University of AveiroAveiro, Portugal

**Keywords:** *Escherichia coli*, antibiotic resistance, fecal pollution, water quality, Microbial source tracking

## Abstract

The aim of this study was to examine antibiotic resistance (AR) dissemination in coastal water, considering the contribution of different sources of fecal contamination. Samples were collected in Berlenga, an uninhabited island classified as Natural Reserve and visited by tourists for aquatic recreational activities. To achieve our aim, AR in *Escherichia coli* isolates from coastal water was compared to AR in isolates from two sources of fecal contamination: human-derived sewage and seagull feces. Isolation of *E. coli* was done on Chromocult agar. Based on genetic typing 414 strains were established. Distribution of *E. coli* phylogenetic groups was similar among isolates of all sources. Resistances to streptomycin, tetracycline, cephalothin, and amoxicillin were the most frequent. Higher rates of AR were found among seawater and feces isolates, except for last-line antibiotics used in human medicine. Multi-resistance rates in isolates from sewage and seagull feces (29 and 32%) were lower than in isolates from seawater (39%). Seawater AR profiles were similar to those from seagull feces and differed significantly from sewage AR profiles. Nucleotide sequences matching resistance genes *bla*_TEM_, *sul1*, *sul2*, *tet*(A), and *tet*(B), were present in isolates of all sources. Genes conferring resistance to 3rd generation cephalosporins were detected in seawater (*bla*_CTX-M-1_ and *bla*_SHV-12_) and seagull feces (*bla*_CMY-2_). Plasmid-mediated determinants of resistance to quinolones were found: *qnrS1* in all sources and *qnrB19* in seawater and seagull feces. Our results show that seawater is a relevant reservoir of AR and that seagulls are an efficient vehicle to spread human-associated bacteria and resistance genes. The *E. coli* resistome recaptured from Berlenga coastal water was mainly modulated by seagulls-derived fecal pollution. The repertoire of resistance genes covers antibiotics critically important for humans, a potential risk for human health.

## Introduction

Fecal contamination in aquatic environments contributes to the spread of human pathogens along with the dissemination of antibiotic-resistant bacteria. Overexposure to antibiotics is leading to increasing levels of resistance in the human (and other animals) commensal microbiota (Austin et al., 1999). The resistomes of fecal bacteria, once in environmental settings, contribute with antibiotic resistance (AR) genes to non-resistant indigenous microorganisms (Aminov, 2011; Tacão et al., 2014). In aquatic systems the cycle may include subsequent transmission of AR to human-associated bacteria (Devirgiliis et al., 2011; Figueira et al., 2011). At the same time, enrichment in AR bacteria is promoted by the presence of antimicrobials or other contaminants in the environment. This facts reinforce the need to identify the sources of antibiotic-resistant bacteria in aquatic environments of human usage (Rosewarne et al., 2010; Gomez-Alvarez et al., 2012).

*Escherichia coli* is a natural member of the intestinal microbiota of human and other homeothermic animals and is a fecal indicator bacteria of election (see review by Harwood et al., 2014). Multi-resistant strains are found in environments such as coastal waters. Although *E. coli* is considered harmless and commensal, several pathotypes have been recognized as significant human pathogens, comprising strains implicated in gastro-intestinal, urinary, or respiratory infections (Kaper et al., 2004). *E. coli* strains can be assigned to four main phylogenetic groups: A, B1, B2 and D (Clermont et al., 2000). Extraintestinal pathogenic bacteria usually belong to groups B2 and D, precisely those that include strains with a larger diversity of virulence factors. The commensal strains belong to groups A and B1 (Picard et al., 1999; Johnson et al., 2001).

In recent years, *E. coli* has been recognized as a major player in the dissemination of AR (Henriques et al., 2006a; Zhao and Dang, 2012). Resistance genotypes so far characterized include genes for resistance to last-line antibiotics such as 3rd generation cephalosporins and fluoroquinolones (Alouache et al., 2012; Wellington et al., 2013; Tacão et al., 2014). To some extent, similar resistance phenotypes and genotypes are shared by strains exposed to similar environmental pressures (Wicki et al., 2011). Considering this fact, some animal hosts may favor the preferential development of certain genotypes.

Seagulls readily utilize food sources of human origin, especially garbage, which may contribute to the high levels of AR associated to their microbiota (Bonnedahl et al., 2009; Poirel et al., 2012). Multi-resistant phenotypes are found in *E. coli* strains isolated from seagull droppings in different geographic areas (Gionechetti et al., 2008; Bonnedahl et al., 2009; Dolejska et al., 2009; Poirel et al., 2012). Genes encoding ESBLs (extended spectrum beta-lactamases), such as *bla*_CTX−M−1_ and *bla*_CTX−M−15_ (Bonnedahl et al., 2009; Dolejska et al., 2009; Simões et al., 2010), and resistance to quinolones (like the plasmid-mediated *qnrS*) (Literak et al., 2010; Vredenburg et al., 2014) integrate the repertoire of seagull's resistome.

Freshwater environments are well-recognized reactors for the dissemination and evolution of AR (Baquero et al., 2008; Figueira et al., 2011; Tacão et al., 2012) but processes occurring in seawater are less understood. The presence of AR strains of *E. coli* in coastal water represents a health issue in areas that are used for recreational activities.

Berlenga is an uninhabited island, recently added to the World Network of Biosphere Reserves due to its ecological relevance. Episodes of fecal pollution have been recorded in its coastal water (Araújo et al., 2014) with seagulls confirmed as the main source of fecal contamination. Human-derived sewage (from visiting tourists) was also suggested as a secondary source (Araújo et al., 2014).

Because it is a well-delimited ecosystem, the island constitutes a privileged location to study pathways implicated in dissemination of AR in natural environments. Resistance to 3rd generation cephalosporins or fluoroquinolones, used as front-line drugs for treatment of Gram-negative infections (Cantón and Coque, 2006; Cattoir et al., 2007), deserved special attention.

In this study the focus was put in coastal water and in the specific contribution of the two previously identified sources of fecal pollution. To accomplish our aims, *E. coli* strains were characterized at the phenotypic level with respect to their resistances profiles; at the genotypic level, specific genes were used as indicators for the resistance to last-line use antibiotics. Phenotypic and genotypic data from coastal water strains was compared with data obtained from the strains of each of the two sources of fecal contamination. Main contributions to the water resistome are hypothesized and discussed.

## Materials and methods

### Study site and sample collection

Berlenga Island is the main island of the Berlengas archipelago, located in the Portuguese continental shelf, 5.7 miles northwest of Cape Carvoeiro. The island has a great diversity of habitats, marine species, and avifauna, including a large population of the yellow-legged gull (*Larus* [*cachinnans*] *michahellis*). Recently, Berlengas was added to the UNESCO'S World Biosphere Reserve. Although uninhabited, many tourists visit the island in the summer mainly for recreational activities such as swimming and diving (Araújo et al., 2014). Human-derived sewage is collected on a settlement tank and discharged near the coastline of the island.

Samples of seagull feces, human-derived sewage and seawater from the Berlenga beach were collected as described in Araújo et al. (2014). Sampling was performed every 2 weeks between May and September 2011. Briefly, samples of seagull feces, human-derived sewage and seawater were collected in each sampling date and consisted in: (i) 5 composite samples of seagull feces scattered on the beach; (ii) 250 mL of raw human-derived sewage taken from the effluent of the sanitary infrastructures; and (iii) 2 L of seawater collected about 30 cm below the surface. Samples were collected in sterile containers and kept on ice until processing.

### *E. coli* strains

A total of 939 *E. coli* isolates were retrieved in Chromocult Coliform Agar plates (Merck, Germany) from seagull feces (*n* = 427), sewage (*n* = 170) and seawater (*n* = 342) as described by Araújo et al. (2014). Genetic diversity of the *E. coli* isolates was inspected by BOX-PCR with primers and conditions previously described (Araújo et al., 2014). Fingerprinting analysis allowed the selection of 414 non-clonal isolates, which were included in the present study. From those, 179 isolates were from seagull feces, 69 isolates were from sewage and 166 were from seawater.

### Determination of *E. coli* phylogenetic groups

The triplex PCR developed by Clermont et al. (2000), with primers for genes *chuA* and *yjaA* and for the DNA fragment TSPE4-C2, was used to determine the phylogenetic groups of the *E. coli* strains. Template DNA was obtained by suspending 2 bacterial colonies in 100 μL of sterile water. For each PCR reaction, 1 μL of this suspension was used as template. Appropriate positive and negative controls were included in the assay.

### Antibiotic susceptibility testing

The *E. coli* isolates were tested for susceptibility to 16 antibiotics by disk diffusion method on Mueller-Hinton Agar (Oxoid, Basingstoke, UK) according to the Clinical and Laboratory Standards Institute guidelines (CLSI, 2012). The following disks (Oxoid, UK) were used: ampicillin (10 μg), amoxicillin (10 μg), amoxicillin/clavulanic acid (20/10 μg), piperacillin (100 μg), piperacillin/tazobactam (100/10 μg), cephalothin (30 μg), ceftazidime (30 μg), cefotaxime (30 μg), gentamicin (10 μg), streptomycin (10 μg), imipenem (10 μg), nalidixic acid (30 μg), ciprofloxacin (5 μg), tetracycline (30 μg), chloramphenicol (30 μg) and trimethoprim/sulfamethoxazole (1.25/23.75 μg). *E. coli* ATCC 25922 was used as quality control. Isolates were classified as sensitive, intermediate or resistant according to the CLSI recommendations after 24 h incubation at 37°C. To determine the percentage of resistant strains to each antibiotic and to define resistance phenotypes, intermediate and resistant isolates were subsequently grouped in a same resistant class.

### Antibiotic resistance gene detection

*Escherichia coli* strains displaying resistance and intermediate resistance phenotypes were screened by PCR to detect genes conferring resistance to β-lactams (*bla*_TEM_, *bla*_SHV_, *bla*_CTX−M_, *bla*_IMP_, *bla*_VIM_, *bla*_KPC_, *bla*_OXA−48_, *bla*_GES_, *bla*_AmpC−like_), tetracycline [*tet*(A), *tet*(B)], quinolones (*qnrA*, *qnrB*, *qnrS*) and sulfonamides (*sul1*, *sul2*). The genes tested were chosen among the most prevalent in clinical and environmental *E. coli* isolates. The PCR reactions were performed in a MyCycler Thermal cycler (Bio-Rad, USA). Primers and PCR conditions are presented in Table 1 and Table S1 (Supporting Information). The reaction mixtures (25 μL total volume) consisted of 6.25 μL NZYTaq 2x Green Master Mix (2.5 mM MgCl_2_; 200 μM dNTPs; 0.2 U/μL DNA polymerase) (NZYtech, Portugal), 16.25 μL of ultrapure water, 0.75 μL of each primer (reverse and forward), and 1 μL of cell suspension prepared as described above. The presence of *bla*_AmpC−like_ genes was inspected using a multiplex PCR as described by Dallenne et al. (2010). Negative and positive controls were included in each PCR experiment. The negative control differed from other reaction mixtures by substituting the cell suspension for the same volume of dH_2_O; positive control strains are indicated in Table 1. PCR products were analyzed by electrophoresis on a 1.5% agarose gel and stained with ethidium bromide. All amplicons obtained using primers for *bla*_SHV_, *bla*_CTX−M_, *bla*_AmpC−like_, *qnrA*, *qnrB*, and *qnrS* and PCR products obtained using primers for *bla*_TEM_ from 3rd generation cephalosporins-resistant isolates were sequenced (accession numbers KM094197 to KM094211). For this PCR products were purified with DNA Clean & Concentrator (Zymo Research, USA) following manufacturer's instructions, and used as template in the sequencing reactions. Online similarity searches were performed with the BLAST software at the National Center for Biotechnology Information website.

**Table 1:** **PCR primers and conditions for AR genes amplification**.

**Target**	**Primer sequence (5′–3′)**	**Amplicon size (bp)**	**Program^a^**	**Annealing temperature (°C)**	**References**	**Control strains**
*bla*_OXA-48_	OXA_F:TTGGTGGCATCGATTATCGG OXA_R: GAGCACTTCTTTTGTGATGGC	744	E	55	Poirel et al., 2004	*S. xiamenensis* IR34
*bla*_TEM_	TEM_F: AAAGATGCTGAAGATCA TEM_R: TTTGGTATGGCTTCATTC	425	B	44	Speldooren et al., 1998	*K. pneumoniae* 6T
*bla*_SHV_	SHV_F: GCGAAAGCCAGCTGTCGGGC SHV_R: GATTGGCGGCGCTGTTATCGC	304	B	62	Henriques et al., 2006a	*K. pneumoniae* 2s
*bla*_CTX-M_	CTX_F: SCVATGTGCAGYACCAGTAA CTX_R: GCTGCCGGTYTTATCVCC	652	A	55	Lu et al., 2010	*K. pneumoniae* Kp40
*bla*_IMP_	IMP_F: GAATAGAGTGGCTTAATTGTC IMP_R: GGTTTAAYAAAACAACCACC	232	B	55	Henriques et al., 2006b	*K. pneumoniae* KP99c196
*bla*_VIM_	VIM_F: GATGGTGTTTGGTCGCATATCG VIM_R: GCCACGTTCCCCGCAGACG	475	B	58	Henriques et al., 2006a	*P. aeruginosa* NTU-39/00
*bla*_KPC_	KPC_F: CATTCAAGGGCTTTCTTGCTGC KPC_R: ACGACGGCATAGTCATTT	538	B	55	Dallenne et al., 2010	*K. oxytoca* Ko25
*bla*_GES_	GES_F: AGTCGGCTAGACCGGAAAG GES_R: TTTGTCCGTGCTCAGGAT	399	D	57	Dallenne et al., 2010	*K. pneumoniae* 22K9
*tet*(A)	tetA_F: GCTACATCCTGCCTTC tetA_R: GCATAGATCGGAAGAG	211	C	53	Nawaz et al., 2006	*E. coli* M.I.10.2
*tet*(B)	tetB_F: TCATTGCCGACCTCAG tetB_R: CCAACCATCACCATCC	391	C	53	Nawaz et al., 2006	*E. coli* M.I.10.1
*qnr*A	qnrA_F: TTCTCACGCCAGGATTTG qnrA_R: CCATCCAGATCGGCAAA	521	C	53	Guillard et al., 2011	*K. pneumoniae* Kp 51
*qnr*B	qnrB_F: GGMATHGAAATTCGCCACTG qnrB_R: TTYGCBGYYCGCCAGTCG	261	C	53	Cattoir et al., 2007; Guillard et al., 2011	*K. pneumonia* Kp1
*qnr*S	qnrS_F: GCAAGTTCATTGAACAGGGT qnrS_R: TCTAAACCGTCGAGTTCGGCG	428	C	54	Cattoir et al., 2007	*K. oxytoca* Ko25
*sul1*	sul1_F: CTGAACGATATCCAAGGATTYCC sul1_R: AAAAATCCCACGGRTC	239	C	50	Heuer and Smalla, 2007	*A. media*
*sul2*	sul2_F: GCGCTCAAGGCAGATGGCAT sul2_R: GCGTTTGATACCGGCACCCG	293	C	60	Henriques et al., 2006a	*E. coli* A237

a *A, B, C, D, and E represent different PCR programs (see Table S1 for details)*.

The genomic contexts of both genes were determined by PCR using previously described primers and conditions (Saladin et al., 2002; Eckert et al., 2006; Tacão et al., 2012). Primers targeted either *bla*_CTX−M−1_ or *bla*_CMY−2_ and genes previously found in the vicinity of these resistance genes (i.e., IS*ECP1* for *bla*_CTX−M−1_ and *bla*_CMY−2_; *ORF477* for *bla*_CTX−M−1_) (Saladin et al., 2002; Eckert et al., 2006; Tacão et al., 2012). PCR fragments were sequenced as described above.

### Statistical analysis

AR phenotypes for each isolate were converted into a numeric code for each antibiotic: 0 signifying susceptibility, 1 representing intermediate resistance and 2 representing resistance. Principal Component Analysis (PCA, using a covariance matrix model) was used to explore AR patterns, reducing the multidimensional data matrix to a bidimensional biplot fit for interpretation, as performed in suchlike studies (Parveen et al., 1997; Su et al., 2012; Pereira et al., 2013). One-way analyses of variance (ANOVA) were performed on the PCA sample scores (principal components 1 and 2—PC1 and PC2) to assess significant differences (*p* ≤ 0.05) in the AR profiles of *E. coli* isolates among sampling sources or phylogenetic groups. When applicable, *post-hoc* Tukey tests followed ANOVA tests. ANOVAs and PCA were conducted using Minitab and CANOCO for Windows version 4.5 (Scientia Ltd., UK), respectively.

## Results

### Phylogenetic diversity of *E. coli* isolates

**Figure 1:**
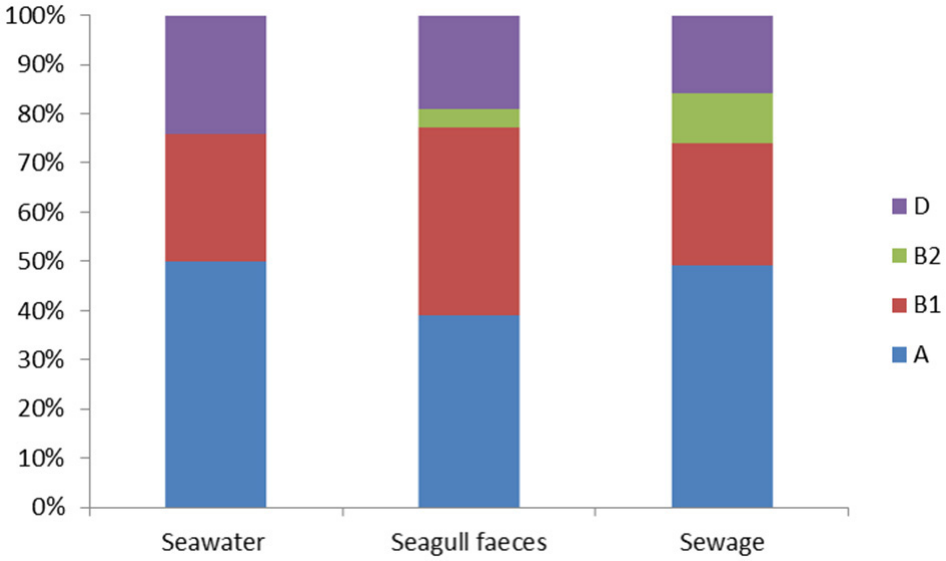
**Distribution of the *E. coli* phylogenetic groups (A, B1, B2 and D) among seawater (*n* = 166), seagull feces (*n* = 179) and human-derived sewage (*n* = 69)**.

The set of 414 *E. coli* isolates were assigned to the main phylogenetic groups: A, B1, B2, and D (Figure 1). Overall, group A was the most prevalent (45% of the total number of isolates) followed by group B1 (31%), group D (21%) and group B2 (3%). Considering the different sampling sources (e.g., seawater, seagull feces and human-derived sewage) distribution among phylogenetic groups was rather similar, except for group B2 isolates that were only detected in sewage (10% of the total number of isolates retrieved from this source) and in seagull feces (4%).

### Antibiotic resistance profiles

**Figure 2:**
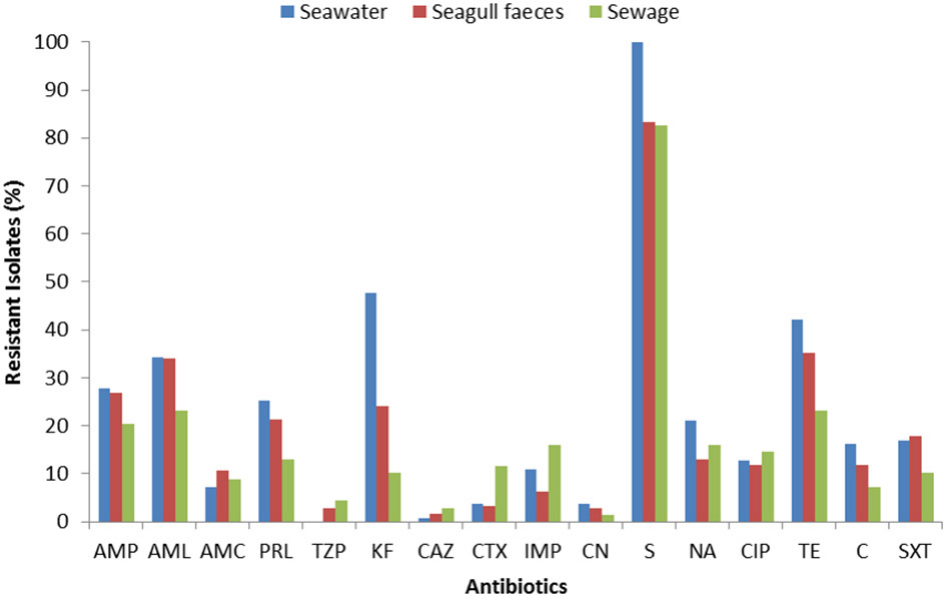
**Total resistance frequencies to 16 antibiotics of *E. coli* strains isolated from seawater (*n* = 166), seagull feces (*n* = 179) and human-derived sewage (*n* = 69)**. AMP, ampicillin; AML, amoxicillin; AMC, amoxicillin/clavulanic acid; PRL, piperacillin; TZP, piperacillin/tazobactam; KF, cephalothin; CAZ, ceftazidime; CTX, cefotaxime; IMP, imipenem; CN, gentamicin; S, streptomycin; NA, nalidixic acid; CIP, ciprofloxacin; TE, tetracycline; C, chloramphenicol; SXT, sulfamethoxazole/trimethoprim.

The susceptibility patterns of the isolates to 16 antibiotics are shown in Figure 2. Resistance (or intermediate resistance) was detected to all antibiotics tested and 94% of the isolates were resistant to at least one of the antibiotics. The most prevalent AR was toward streptomycin (83–100%), followed by tetracycline and cephalothin in seawater (48 and 42% respectively), and tetracycline and amoxicillin in the fecal sources (35 and 34% in seagull feces and 23% for both antibiotics in sewage). Isolates were more susceptible to imipenem, 3rd generation cephalosporins, gentamicin and the combination piperacillin/tazobactam.

For the majority of the antibiotics tested (e.g., penicillins, cephalothin, tetracycline), resistance rates were higher in isolates from seawater and seagull feces (Figure 2). Resistance to imipenem, 3rd generation cephalosporins and ciprofloxacin was most common in sewage. Multi-resistance levels (i.e., resistance to antibiotics of at least three different classes) were higher among isolates recovered from seawater (39%), followed by isolates from seagull feces (32%) and sewage (29%).

**Figure 3:**
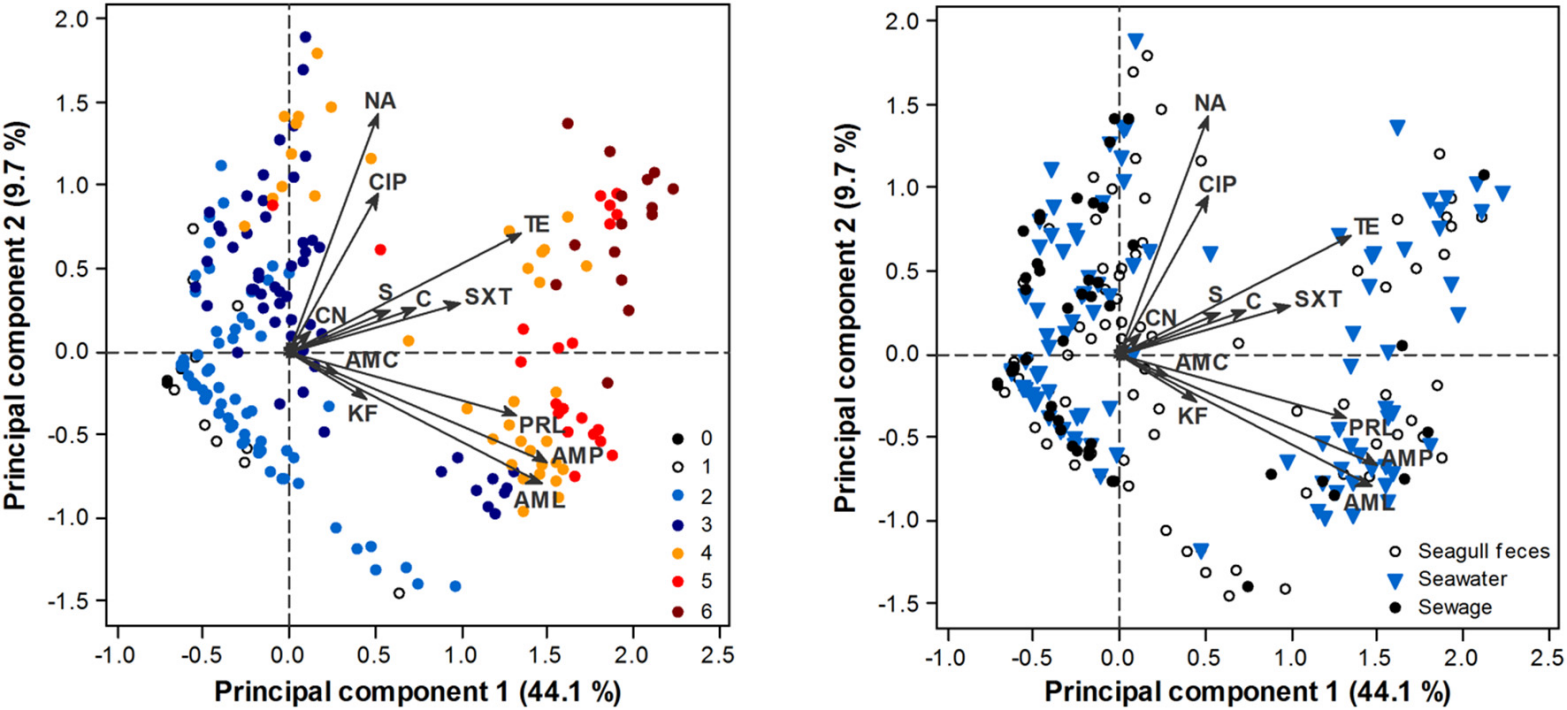
**PCA biplot of *E. coli* isolate scores in terms of their AR profile**. Arrows represent antibiotics: AMP, ampicillin; AML, amoxicillin; AMC, amoxicillin/clavulanic acid; PRL, piperacillin; TZP, piperacillin/tazobactam; KF, cephalothin; CAZ, ceftazidime; CTX, cefotaxime; IMP, imipenem; CN, gentamicin; S, streptomycin; NA, nalidixic acid; CIP, ciprofloxacin; TE, tetracycline; C, chloramphenicol; SXT, sulfamethoxazole/trimethoprim. TZP, CAZ, CTX, IMP have little influence in the ordination, thus respective arrows are not perceptible. Scores are depicted according to the number of antibiotic classes that isolates are resistant to (0–6; **left panel**) or source of origin (**right panel**).

PCA analysis confirmed a clear AR gradient, with less resistant isolates (isolates resistant to a lower number of antibiotics) consistently displaying lower PC1 scores (i.e., being located at the left of the biplot) and more resistant isolates displaying higher PC1 scores (i.e., being located at the right of the biplot) (Figure 3). Although resistant and multi-resistant isolates were found in all sampling sources, significant differences in PC1 scores were found among sources [ANOVA: *F*_(2, 411)_ = 3.7, *p* = 0.025]. This was mostly due to a significantly higher PC1 score in seawater isolates comparatively to sewage isolates (Tukey test, *p* < 0.05). No significant differences in terms of the AR profile were found across phylogenetic groups, either for PC1 [ANOVA: *F*_(2, 397)_ = 1.59, *p* = 0.206] or PC2 [ANOVA: *F*_(2, 397)_ = 0.43, *p* = 0.649]; group B2 was excluded from this analysis due to low representativeness.

A total of 126 different resistance (or intermediate resistance) phenotypes were observed but most (*n* = 86) were represented by only one isolate. Resistance to streptomycin was the most common phenotype, identified in 90 isolates, followed by resistance to streptomycin and tetracycline (30 isolates) and streptomycin and cephalothin (27 isolates). Several multi-resistance phenotypes were detected. Among these, 10 phenotypes included 8 antibiotics and 9 phenotypes included 9 antibiotics. Twenty-four phenotypes (Table 2) were common to seawater and to at least one of the putative pollution sources (i.e., sewage and seagull feces). From these, 45% (*n* = 11) were common to seawater and seagull feces but were absent from human-derived sewage, while only 8% (*n* = 2) of the phenotypes were exclusively detected in both seawater and sewage.

**Table 2:** **AR phenotypes detected in seawater and in at least one of the putative pollution sources (i.e., seagull feces and sewage)**.

**Phenotypes**	**Number of isolates**			
	**Seawater**	**Seagull feces**	**Sewage**	**Total (% of the total number of isolates)**
S	31	39	20	90 (21.7)
S, TE	14	13	3	30 (7.2)
KF, S	22	5	0	27 (6.5)
AML, KF, S	6	4	1	11 (2.7)
IPM, S	4	4	0	8 (1.9)
S, NA	4	1	1	6 (1.4)
AMP, AML, PRL, KF, S, TE	2	3	1	6 (1.4)
AMP, AML, PRL, KF, S, TE, STX	2	4	0	6 (1.4)
AML, S	1	3	1	5 (1.2)
AMP, S	1	0	3	4 (1.0)
S, NA, TE	2	1	1	4 (1.0)
AMP, AML, PRL, S, TE	2	1	1	4 (1.0)
AMC, S	1	2	0	3 (0.7)
S, TE, STX	2	1	0	3 (0.7)
AML, KF, S, TE	2	1	0	3 (0.7)
AMP, AML, KF, S	1	2	0	3 (0.7)
AMP, AML, PRL, S	1	2	0	3 (0.7)
AMP, AML, PRL, S, TE, STX	2	1	0	3 (0.7)
AMP, AML, AMC, PRL, KF, S, TE, C, STX	1	1	1	3 (0.7)
AMP, AML, PRL, KF, S, NA, CIP, TE, C, STX	2	1	0	3 (0.7)
AMP, AML, AMC, KF, S	0	2	0	2 (0.5)
KF, S, TE	1	1	0	2 (0.5)
AMP, AML, PRL, KF, S, TE, C, STX	1	1	0	2 (0.5)
AMP, AML, AMC, PRL, IPM, S, TE, C, STX	1	0	1	2 (0.5)

*The number of isolates from each sampling source displaying each phenotype is presented*.

*AMP, ampicillin; AML, amoxicillin; AMC, amoxicillin/clavulanic acid; PRL, piperacillin; KF, cephalothin; IPM, imipenem; S, streptomycin; NA, nalidixic acid; CIP, ciprofloxacin; TE, tetracycline; C, chloramphenicol; STX, thrimethoprim/sulfamethoxazole*.

### Detection of antibiotic resistance genes

The resistance genes elected for this study were detected in 157 isolates out of 390 displaying a resistance phenotype (Table 3). The most commonly detected genes were *bla*_TEM_, *sul1*, *sul2*, *tet*(A), and *tet*(B), which were prevalent in 6 out of 8 sampling moments (data not shown). The *bla*_TEM_ gene (*bla*_TEM−1_ according to sequence analysis of representative isolates) was more prevalent in isolates from seawater (69% of 68 isolates resistant to penicillins) than in isolates from seagull feces (38% of) or sewage (24%). Both *tet*(A) and *sul2* were commonly detected in seawater (69 and 64%, respectively) and seagull feces (70 and 60%), but were less frequent in sewage (31 and 43%). Eighty six isolates displaying resistance to quinolones were tested for the presence of *qnr* determinants. Among these, *qnrS* (identified as *qnrS1* by sequence analysis) was the most prevalent, being more frequent in seagull feces (17% of 30 resistant isolates). This gene was detected in 5 out of 8 sampling dates. Gene *qnrB* (*qnrB19* according to sequence analysis) was detected in two sampling dates in isolates from seawater and seagull feces and *qnrA* was not detected. Gene *bla*_SHV−12_ was detected in one isolate from seawater. Genes *bla*_CTX−M_ and *bla*_AmpC−like_ were detected in one isolate each, from seawater and seagull feces respectively. Sequencing analysis revealed 100% identity with *bla*_CTX−M−1_ and *bla*_CMY−2_. The genomic contexts of both genes were determined and results revealed genomic contexts identical to the ones previously described in clinical isolates (Kang et al., 2006; Tacão et al., 2012): IS*ECP1* was detected upstream of both genes and *ORF477* was identified downstream *bla*_CTX−M−1_. The genes encoding resistance to carbapenems (i.e., *bla*_OXA−48_, *bla*_IMP_, *bla*_VIM_, *bla*_GES_ and *bla*_KPC_) were not detected in any of the *E. coli* isolates studied.

**Table 3:** **Prevalence of resistance genes detected in the *E. coli* strains according to the isolation source**.

**Antibiotics**	**Genes**	**Seawater**		**Seagull feces**		**Sewage**	
		**Resistant isolates**	**Positive PCR results**	**Resistant isolates**	**Positive PCR results**	**Resistant isolates**	**Positive PCR results**
3rd generation cephalosporins and/or penicillins	*bla*_TEM_	68^a^	47 (69%)	82^a^	31 (38%)	33^a^	8 (24%)
	*bla*_SHV-12_		1 (2%)		0		0
	*bla*_CTX-M-1_	6^b^	1 (17%)	8^b^	0	8^b^	0
	*bla*_CMY-2_		0		1 (13%)		0
Quinolones	*qnrB19*	39	1 (3%)	30	2 (7%)	17	0
	*qnrS1*		2 (5%)		5 (17%)		1 (6%)
Tetracycline	*tet*(A)	70	48 (69%)	63	44 (70%)	16	5 (31%)
	*tet*(B)		22 (31%)		15 (24%)		6 (38%)
Sulfonamide + trimethoprim	*sul1*	28	8 (29%)	32	8 (25%)	7	2 (29%)
	*sul2*		18 (64%)		19 (60%)		3 (43%)

a *bla_TEM_ and bla_SHV_ were inspected in all penicillin-resistant isolates (n = 183), 22 of which were also resistant to 3rd generation cephalosporins*.

b *bla_CTX-M_ and bla_CMY_ were inspected in isolates displaying resistance both to penicillins and 3rd generation cephalosporins (n = 22)*.

A total of 27 different AR genotypes were observed. Among these, 7 included only 1 resistance gene, 7 included 2 genes and 8 included 3 genes. Four genotypes included 4 genes: *bla*_TEM_-*tet*(A)-*sul1-sul2*, detected in 4 isolates from seawater and 5 isolates from seagull feces; *bla*_TEM_-*tet*(B)-*sul1-sul2*, detected in 2 isolates from seawater and 2 isolates from sewage; *bla*_TEM_-*bla*_SHV−12_-*tet*(A)-*sul2* and *bla*_TEM_-*bla*_CTX−M−1_-*tet*(A)-*qnrS1*, each detected in 1 seawater isolate. The genotype *bla*_TEM_-*tet*(B)-*qnrS1*-*sul1-sul2* was detected in 1 isolate from seagull feces. Fourteen genotypes were shared between seawater and at least 1 of the putative pollution sources (Table 4). From these, 50% (*n* = 7) were shared between seawater and seagull feces, exclusively. Only 1 genotype was common to seawater and sewage, and absent from seagull feces.

**Table 4:** **AR genotypes detected in seawater and in at least one of the putative pollution sources (i.e., seagull feces and sewage)**.

**Genotype**	**Seawater**	**Seagull feces**	**Sewage**	**Total (%)**
*tet*(A)	21	20	3	44 (11.3)
*bla*_TEM_, *tet*(A)	13	6	2	21 (5.4)
*tet*(B)	7	8	2	17 (4.4)
*bla*_TEM_, *tet*(B)	7	3	2	12 (3.1)
*bla*_TEM_, *tet*(A), *sul*2	4	5	0	9 (2.3)
*bla*_TEM_, *tet*(A), *sul1*, *sul2*	4	5	0	9 (2.3)
*bla*_TEM_	3	4	1	8 (2.1)
*bla*_TEM_, *tet*(B), *sul2*	4	1	0	5 (1.3)
*sul2*	1	3	0	4 (1.0)
*bla*_TEM_, *tet*(B), *sul1*, *sul2*	2	0	2	4 (1.0)
*tet*(A), *sul2*	1	2	0	3 (0.8)
*bla*_TEM_, *sul2*	1	1	1	3 (0.8)
*bla*_TEM_, *tet*(A), *qnrS1*	1	2	0	3 (0.8)
*bla*_TEM_, *tet*(A), *tet*(B)	1	1	0	2 (0.5)

*The number of isolates from each sampling source displaying each phenotype is presented*.

## Discussion

The threats for human health represented by fecal pollution in aquatic environments depend on whether the source is human or non-human (Field and Samadpour, 2007). Dissemination of AR is certainly a risk factor. The particular characteristics of the Berlenga Island allowed evaluation of the contribution given by human-derived sewage and seagull feces, to the AR patterns found in *E. coli* strains that can be grown and isolated from coastal water samples.

High prevalence of resistance to streptomycin, tetracycline, cephalothin, and amoxicillin was detected in all sources. Results are in agreement with previous studies that demonstrated a wide dissemination of resistance to these antibiotics among *E. coli* isolates from Portuguese clinical settings (Freitas et al., 2014), animals (Radhouani et al., 2009) and aquatic systems (Pereira et al., 2013). For antibiotics mainly used in human medicine such as 3rd generation cephalosporins, ciprofloxacin and imipenem, levels of resistance were low. An uncommon phenotype combining resistance to imipenem and streptomycin and susceptibility to penicillins was detected in eight strains, six of which were isolated from sewage. Genes encoding carbapenemases were not detected in these strains, an indication that non-enzymatic mechanisms, such as the expression of efflux pumps or porin loss (Papp-Wallace et al., 2011), may contribute to the phenotype. Concerning 3rd generation cephalosporins, the resistance phenotype was explained only in three out of twenty-two resistant isolates. Novel or uncommon resistance genes may be present in the genomes of the remaining strains. On the other hand, mutations in the promoter and attenuator sequences of the chromosomal *ampC* gene may also contribute to reduced susceptibility to these antibiotics. These mutations have been described in *E. coli* isolates from different sources, including aquatic systems (Mataseje et al., 2009).

Levels of AR in coastal water isolates were expected to be low, considering the moderate human impact and the limits to survival imposed to *E. coli* by water salinity. However, experimental data revealed resistance levels similar to those previously reported for polluted freshwater (Ham et al., 2012; Pereira et al., 2013) or wastewater (Figueira et al., 2011). Multi-resistance levels were higher in coastal water (39%) than in the two sources of fecal contamination (32% for seagull feces and 29% found in human sewage).

Genes frequently hosted by clinical isolates and sharing similar genomic contexts (Saladin et al., 2002; Eckert et al., 2006), were also hosted by seawater isolates. Examples are ESBL-encoding genes such as *bla*_CTX−M−1_ and *bla*_SHV−12_ or plasmid-mediated quinolone resistance genes (e.g., *qnrB19*). It is to point that dissemination of these genes or related ones is a matter of serious concern in a scenario of lack of innovation in what concerns antimicrobial therapies. In fact, 3rd generation cephalosporins and fluoroquinolones are front-line therapeutics for the treatment of Gram-negative infections (Cantón and Coque, 2006; Cattoir et al., 2007).

AR features of seawater *E. coli* strains are mainly dependent on their animal source (Wicki et al., 2011). At the same time, *E. coli* strains are able to adapt to non-human environments, a process that implies changes in their genetic background (van Elsas et al., 2011). Adaptation to the more stressful saline conditions of coastal waters may favor strains with genotypes that are more efficient to deal with other stresses.

The results strongly suggest that the *E. coli* resistome found in coastal water was mainly impacted by fecal pollution from seagulls. The AR patterns found on seagull feces and seawater shared abundant common elements, while differing significantly from the resistance patterns found on sewage. Differences were not attributable to a different distribution in terms of phylogenetic groups. Based on these observations it is possible to hypothesize that the enteric environment of seagulls positively selects *E. coli* strains more prone to survive in marine environments. The same data allows proposing resistome analysis as a microbial source-tracking tool (see Harwood et al., 2014). Such approach has been previously tested, using almost exclusively data resulting from the analysis of resistance phenotypes (Parveen et al., 1997; Field and Samadpour, 2007; Carroll et al., 2009). Data on resistance genotypes add valuable information for the identification of the contamination source. The resistance gene pool in the analyzed sources was quite stable along the period of the study, except for the sporadic detection of infrequent genes (data not shown). Our recommendation is toward the use of genotypic data only in combination with other data. Our data further confirmed the conclusion of Araújo et al. (2014) establishing seagulls as the main source of fecal pollution on Berlenga Island.

This study highlights the risks associated to seagull-derived pollution. This is a critical aspect since wild life-derived pollution is frequently neglected due to the assumption that host-specificity of pathogens (Field and Samadpour, 2007) is a preventive barrier, that makes difficult if not impossible, the establishment on a new host. Indeed, the contribution of seagull fecal bacteria to AR dissemination constitutes an undeniable risk to humans, especially because of the close contact between these birds and human waste. Previous studies have confirmed seagulls as a reservoir of AR (Gionechetti et al., 2008; Bonnedahl et al., 2009; Dolejska et al., 2009). In our study, besides the high level of multi-resistance in seagull feces, we retrieved isolates carrying genes conferring resistance to antibiotics that are critically important for human medicine. For example, the prevalence of isolates carrying the *qnrS1* gene was higher in seagull feces than in sewage. This plasmid-encoded gene was previously identified in isolates from seagull feces (Literak et al., 2010) and is frequently found in human pathogens (Cattoir et al., 2007). Likewise, we detected the presence of *qnrB19* in seawater and seagull feces, a gene which was previously detected in animal and human isolates (Dolejska et al., 2013). Moreover, the plasmid-encoded gene *bla*_CMY−2_, conferring resistance to 3rd generation cephalosporins, was detected in seagull feces and in a genomic context identical to the one found in clinical isolates (Verdet et al., 2009). Poirel et al. (2012) reported a high proportion of CMY-2-positive strains among resistant *E. coli* isolated from seagull feces in Miami Beach. So, data obtained in different contexts indicate seagulls as important vectors of antibiotic resistant bacteria.

A previous study in the same Island (Radhouani et al., 2009), showed that all isolates were susceptible to carbapenems and 3rd generation cephalosporins. These antibiotics are mainly used in human medicine (Cantón and Coque, 2006; Papp-Wallace et al., 2011) and resistance to them is known to be still limited in unpolluted or moderately polluted environments (Henriques et al., 2012; Tacão et al., 2012). As reported here, resistance to these antibiotics was most common in human-derived sewage, but its presence on seagull feces isolates suggests an undesirable progress in terms of resistance dissemination. Seagulls based on the Island fly to urban areas on the coast for feeding. Like suggested for other geographic regions (Bonnedahl et al., 2009), human activities may considerably influence the AR patterns associated to seagulls in remote areas, such as Berlenga. In fact, it is a matter of concern that levels of resistance to 3rd generation cephalosporins have been consistently increasing in isolates from clinical settings in Portugal (ECDC, 2013). For example, gene *bla*_CMY−2_, which was here detected in one isolate from seagull feces, has been detected in *E. coli* from clinical samples in Portuguese health care settings (Freitas et al., 2014).

## Conclusions

This study confirms seawater as a relevant reservoir of *E. coli* strains adapted to marine environments and carrying AR determinants that are common to clinical strains. It is evidenced that the seawater *E. coli* resistome in Berlenga is mainly modulated by seagulls-derived pollution. This fact confirms seagulls as a main spread vehicle of AR bacteria to coastal water. Occurrence of high levels of multi-resistance and of clinically relevant genes underlies the need to carefully consider the human health risks subjacent to seagull-derived pollution. The assessment of these risks is essential for an effective management of coastal water used for recreational activities. Lastly, we believe that this study—along with correlated data from previous studies—has succeeded in showing that AR could be used as a tool to aid in the identification of the fecal sources of contamination.

### Conflict of interest statement

The authors declare that the research was conducted in the absence of any commercial or financial relationships that could be construed as a potential conflict of interest.
