# Supplementary material for: Seawater is a reservoir of multi-resistant Escherichia coli, including strains hosting plasmid-mediated quinolones resistance and extended-spectrum beta-lactamases genes
Source: Front Microbiol. 2014 Aug 20;5:426. doi: 10.3389/fmicb.2014.00426 (PMC4138442; doi:10.3389/fmicb.2014.00426)
Supplement: Supplementary file 1 [file DataSheet1.DOCX]

***Supplementary Material***

**Seawater is a reservoir of multi-resistant *Escherichia coli*, including strains hosting plasmid-mediated quinolones resistance and extended-spectrum beta-lactamases genes**

**Marta Alves, Anabela Pereira, Susana Araújo, Bruno B. Castro, António Correia and Isabel Henriques***

Department of Biology & CESAM, University of Aveiro, Aveiro, Portugal

*** Correspondence:** Isabel Henriques, Department of Biology & CESAM, University of Aveiro, campus Universitário de Santiago, Aveiro, 3810-193 Aveiro, Portugal.

[ihenriques@ua.pt](mailto:ihenriques@ua.pt)

1. **Supplementary Figures and Tables**

## Supplementary Tables

**Supplementary Table 1.** PCR programs (A to E) used for amplification of antibiotic resistance genes.

|  | **Temperature** | **A** | **B** | **C** | **D** | **E** |
| --- | --- | --- | --- | --- | --- | --- |
| 1^st^ step | 94 ºC | 2 min | 5 min | 5 min | 10 min | 5 min |
|  | 94ºC | 15 sec | 30 sec | 30 sec | 40 sec | 30 sec |
| 2^nd^ step^a^ | Annealing temperature | 30 sec | 30 sec | 30 sec | 40 sec | 30 sec |
|  | 72ºC | 45 sec | 1 min | 30 sec | 1 min | 1 min |
| 3^rd^ step | 72ºC | 10 min | 7 min | 7 min | 7 min | 7 min |

^a^The second step corresponds to 30 cycles (programs A to D) or 35 cycles (program E).
